# Is Stem Cell Therapy the New Savior for Cerebral Palsy Patients? A Review

**DOI:** 10.7759/cureus.10214

**Published:** 2020-09-02

**Authors:** Varun Vankeshwaram, Ankush Maheshwary, Divya Mohite, Janet A Omole, Safeera Khan

**Affiliations:** 1 Medicine, California Institute of Behavioral Neurosciences & Psychology, Fairfield, USA; 2 Medicine, Zaporozhye State Medical University, Zaporozhye, UKR; 3 Neurology, California Institute of Behavioral Neurosciences & Psychology, Fairfield, USA; 4 Medicine, Government Medical College, Amritsar, IND; 5 Internal Medicine, California Institute of Behavioral Neurosciences & Psychology, Fairfield, USA

**Keywords:** cerebral palsy, stem cell therapy

## Abstract

Cerebral Palsy (CP) is one of the foremost causes of childhood motor disability and disrupts the individual's development and ability to function. Several factors contribute to the development of CP such as preterm delivery, low birth weight, infection/inflammation, and additional pregnancy complications, both in preterm and term infants. As there is no specific treatment for CP, rehabilitation is the current option for the management of patients. The serious nature of this condition creates deficits that last a lifetime.

We collected studies that were published in the past 10 years, using PubMed as our main database. We chose studies that were relevant to CP and stem cell therapy. We mainly focused on various types of stem cells that can be used in treatment, mechanism of action (MOA) of stem cells, routes, dosage, and adverse effects, their efficacy, and safety in CP patients. Of all the 38 studies we reviewed, we found that five articles discussed the utilization of human umbilical cord blood [hUCB], four articles discussed autologous bone marrow stem cells, and one discussed allogeneic umbilical cord blood usage. One article discussed neural stem-like cells (NSLCs) derived from bone marrow and the remaining 27 articles were about CP and its treatment.

We reviewed detailed information about the possible stem cell therapies and their benefits in patients with CP. We found that immune modulation is the major mechanism of action of stem cells, and among all the types of stem cells. Autologous umbilical cord mesenchymal stem cells appear to be safe and most effective in treatment compared to other stem cell treatments. Among all symptoms, motor symptoms are best corrected by stem cell therapy. Still, it did not show any marked improvement in treating other symptoms like speech defects, sensory or cognitive defects, or visual impairment.

## Introduction and background

Cerebral palsy (CP) is a heterogeneous group of conditions involving permanent non-progressive motor dysfunction that affects muscle tone, posture, and movement-related symptoms characterized by spasticity, dyskinesia, and ataxia [1]. Eighty percent of children affected with CP show spasticity as a prominent feature [1]. This is accompanied with other symptoms like pain (50% to 75%), intellectual disability (50%), speech-language disorders (40% to 60%), bladder control problems (30% to 60%), visual impairment (30% to 50%), epilepsy (25% to 45%), behavior disorder (25% to 40%), hip displacement (30%), sleep disorder (20%), and drooling (20%) [2,3]. At least one in four children with CP have emotional and behavioral issues like attention-deficit/hyperactivity disorder, conduct disorders, anxiety, and depression [4]. Quality of life (QOL) is severely impaired in CP patients [5]. Incidence of CP is about two to three in 1000 live births; increased survival of very premature patients might be increasing the incidence of CP [1,6,7]. The perinatal period is the most vulnerable period for the damage; approximately 92% of cases of CP are affected in this period [8]. Major risk factors that can lead to brain injury are preterm birth, perinatal infection (specially chorioamnionitis), intrauterine growth restriction, use of preterm antibiotics before rupturing of membranes, acidosis or asphyxia, and multiple gestations [9,10]. Intrapartum hypoxia believed to be a major cause attributes only to less than 10% cases [9]. Head injury and infection are the causes of CP occur at an older age in about 8% of patients [8]. Despite discussion about many risk factors, 80% of cases have no specific cause and are considered idiopathic [11].

The diagnosis of CP is clinical and usually made between 12 and 24 months of age when there are clinical findings of impaired movement, posture or balance are noticeable, but with the use of perinatal ultrasonography and post-birth magnetic resonance imaging (MRI), the diagnosis can be made as early as six months of age (corrected for prematurity) [5,11]. There is no single test that rules in or rules out CP; hence definitive diagnosis is usually made by serial examinations and supplementary information provided by history and imaging studies like MRI, findings include hypoxic-ischemic lesions (example: periventricular leukomalacia), cortical malformations, and lesions of the basal ganglia [12]. If perinatal imaging studies, such as fetal anatomy surveys or newborn transcranial ultrasonography, do not show a cause for the clinical findings, neuroimaging may be obtained like magnetic resonance imaging (MRI) is the recommended imaging modality and is preferable to computed tomography given its higher specificity (approximately 89%) for identifying intracranial abnormalities [5]. A broad range of abnormalities may be seen on brain imaging in patients with CP, including schizencephaly (clefts in cerebral tissue), hydrocephalus, and periventricular leukomalacia. In one study, only 5% of imaging studies demonstrated findings specific to hypoxic-ischemic injury [13].

Once the diagnosis of CP is established, the Gross Motor Function Classification System is used to evaluate the severity and response to treatment [1]. Integrated management of the child with CP requires a multifaceted team to address the multiple medical, social, psychological, educational, and therapeutic needs. Treatments for the movement disorders associated with CP include systemic and intrathecal muscle relaxants, intramuscular on a botulinum toxin A, selective dorsal rhizotomy, and physical and occupational therapies. However, there has been no complete cure for this illness. Moreover, some of these interventions are the ones that are currently used for treating CP lead to various side effects and unease in these patients. Stem cell therapy is reassuring these days. Recently, stem cell therapy has attracted huge interest as a new therapeutic method for the treatment of CP. The ultimate goal of this therapeutic intervention is to replace damaged tissue with the formation of new tissues by harnessing with the stem cells, which have a good regenerative capacity [14]. Several investigations in animals and humans with CP have demonstrated the positive potential of stem cell transplantation for the treatment of CP disorder. An increasing amount of evidence suggests that umbilical cord blood (UCB) may be used for both early diagnostics and treatment of CP. However, there's still a lack of clarity about this method, and high effort is necessary to create technology as an ideal treatment for this condition. Previously there were few controlled human clinical trials, but now the number of human studies is significantly increasing in support of stem cell therapy. There is still a lot more research to be done in exploring the biology of stem cells, cell differentiation, intrinsic neural cell proliferation, and clear understanding of the mechanism of action of stem cells, dosage, and their side effects in treating CP.

This narrative review aims to report on the current knowledge regarding the clinical use of UCB based on articles from PubMed and clinical trials registered on ClinicalTrials.gov. This review will discuss the scientific background of stem cell therapy for CP, including evidence from current clinical trials. We will be having a detailed discussion regarding various types of stem cells, their mechanism of action, efficacies, dosage, routes of administration, and evaluate the prognosis of CP.

## Review

Methods

We followed the Preferred Reporting Items for Systematic Reviews and Meta-Analyses (PRISMA) guidelines and used PubMed as our main database to search for articles that are relevant to CP and stem cell therapy and explained the use of stem cells for better treatment of CP. We conducted the literature search by using the keywords "Cerebral palsy", "Stem cell therapy."

We found 79 articles altogether that are relevant and peer-reviewed with keywords "stem cell therapy" and "cerebral palsy." We screened the title and abstract relevant to stem cell therapy in CP patients which limited to 41 articles; we further eliminated duplicate articles and finalized 38 articles in total as our references. All the studies that are included were published in English within the last 10 years from the search date (2010-2020). We have not considered any specific geographical location for the study search. Our study results included both the abstract and full-text articles and up to date.

We focused on the potential pathophysiology of CP, mechanism of action of stem cells in the treatment of CP. We concentrated the effect of stem cells mostly on CP and excluded the application of stem cells in the treatment of other medical conditions. We particularly focused on various types of stem cells, the composition of stem cells, and their neurotrophic factors. Animal trials were excluded. All the data was collected ethically and legally. Quality appraisal was done for all the collected studies.

Results

By following the search criteria mentioned in the methods section, we collected a total of 38 articles that were relevant to CP and stem cell therapy [1-38]. Among them, eight were randomized controlled trials (RCTs), 27 were systemic reviews, two meta-analysis, and one was a case report. All the studies included were peer-reviewed and were included after quality assessment using PRISMA for Systematic Reviews and Cochrane bias assessment tool for RCTs.

Discussion

CP is a chronic non-progressive disorder; the incidence of CP is increasing because of the increased survival of preterm infants. Also, prematurity is the most important cause for the occurrence of CP. We did a literature review using PubMed as the major source, collected relevant information regarding different aspects of stem cell therapy for the understanding of better treatment.

Pathophysiology of CP

The major cause for CP is prematurity and also hypoxia-ischemia in the perinatal period insult that leads to the damage to the various areas of the brain, majorly white matter known as periventricular leukomalacia, also germinal matrix hemorrhage with intraventricular extension, also injury to the cortex, basal ganglia, and thalamus [15]. Most of the pathophysiological events are pro-inflammatory markers, free radical injury, cytokine toxicity. All these events mainly affect the promyelinating oligodendrocytes that lead to the periventricular leukomalacia.

Stem Cell Therapy

**Figure 1:**
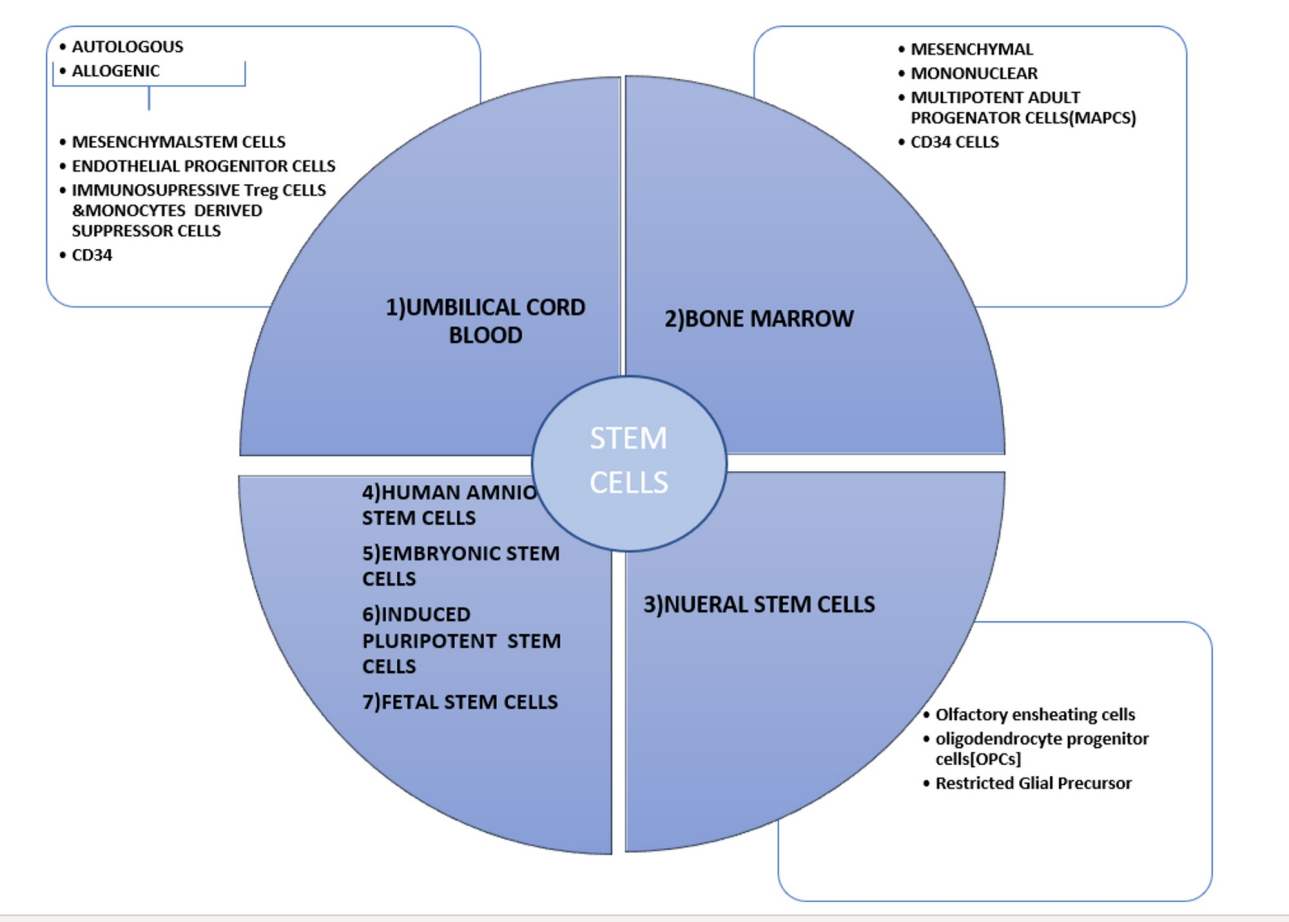
Types and characteristics of different stem cells

Stem cell therapy has accomplished global awareness and attentiveness for the ability to mitigate numerous conditions or disease states. The worldwide figures exhibit that CP is the second most common disease treated with stem cell therapy [16]. It would be important to discuss various aspects of stem cells for a better idea of its implication. Figure 1 below shows the types and characters of the stem cells.

Every single cell type can be used for the prevention and treatment of the brain injury after hypoxic and inflammatory events in the perinatal period, but they vary in different mechanisms, routes, dosages [16,17].

UCB has almost 30 years of clinical utilization [16]. UCB is acidic and comprises of various anti-inflammatory cytokines, growth factors, other metabolites such as amino acids, phosphatidylcholines, acylcarnitine, succinate, glycerol, 3-hydroxybutyrate, and phosphocholine are essential for neurodevelopment [18]. UCB is promptly available during childbirth, can combat long-term cryopreservation, and there is 90% of viable cell restoration post-thaw [16]. Autologous UCB is assumed considerably safer than allogeneic UCB for decades due to the theoretical risk of graft versus host (GVH) disease. Allogenic UCB supposed to be comparatively safe in two clinical trials without any graft versus host (GVH) reaction, but still, more extensive studies must be conducted to clarify the theoretical risk. Whereas in preterm, allogenic cells are presumably better because of limited quantities, also stem cells arising in the background of etiology of CP (example: fetal inflammatory response syndrome), immune immaturity, intrauterine growth restriction, and preeclampsia alter the relative portion of the stem and immune cells [19,20]. Another study shows that EPCs present in UCB may have an augmented benefit using UCB alone. In a study, intravenous infusion of allogeneic human leukocyte antigen (HLA)-unmatched umbilical cord blood (UCB) cells were studied in 80 pediatric patients with CP to understand the safety, efficacy, and associated neurological complications. All the patients received up to six intravenous infusions of AB0/Rh-identical, red blood cell-depleted UCB cells at an average dose of 250 × 10^6^ viable cells per infusion. Patients were followed for three to 36 months, and have noticed that this intervention did not cause any adverse effects with multiple cell infusions [21].

Satellite cells are adult muscle stem cells, precursor cells for skeletal muscle regeneration and growth are markedly reduced in CP may be liable for smaller muscle fiber size and deterioration in muscle growth, and contractile properties. Targeted therapies activating satellite cells help in the better management of contractures in CP patients [22].

Neural stem cells are endogenously produced multipotent cells mostly in the subventricular zone of the developing brain and remain to be found in the adult brain. Endogenous neural stem cells have the potential to self-renew and ability to differentiate into neurons and glial cells [16]. According to the study by Yu et al., the inhabitant stem/progenitor cells vitalized in vivo by neurotrophic factors, hematopoietic growth factors, magnetic stimulation, and exercise might be beneficial to activate neurogenesis and could be used in the treatment of CP [23]. En masse, these findings recommend the opportunity of neurorestorative from endogenous stem cells and helps in the functional brain, should be tested more clinically. 

Thus far, clinical trials showing umbilical mesenchymal stem cells are more beneficial and safe, even compared to bone marrow mesenchymal stem cells (MSCs) [24,25]. However, the identification of the best cell population is still an unresolved issue. Table 1 below shows the benefits of umbilical cord stem cells over bone marrow-derived mesenchymal stem cells.

**Table 1:** Benefits of umbilical cord stem cells over bone marrow-derived mesenchymal stem cells

TYPE OF STEM CELLS	UMBILICAL CORD MESENCHYMAL STEM CELLS	BONE MARROW DERIVED MESENCHYMAL STEM CELLS
SOURCE	UMBILICAL CORD BLOOD	BONE MARROW DERIVED [18]
EFFICIENCY	MORE EFFICIENT	COMPARITIVELY LESS EFFICIENT [18]
ACQUISITION	DIFFICULT TO ACQUIRE	EASY TO ACQUIRE [18]
RISK OF REGESTION	AUTOLOGOUS NO REJECTION, ALLLOGENIC RISK OF REJECTION	NO REJECTION MOSTLY AUTO GENIC [18]
ETHICAL RESTRICTIONS	MAYBE	NO [18]
TELOMERASE ACTIVITY	MORE	LESS [26]
NEUROTROPHIC EFFECT	MORE	LESS [26]

Mechanism of Action of Stem Cells

The pathology of CP is generally regionally confined, non-progressive, and shows limited to cell types, making stem cell therapy as a most promising practice. Stem cells express various mechanisms to combat the damage of hypoxic insult in the brain. The proposed mechanisms of stem cells to treat CP consists of:

Regenerative mechanism: Self-renewal and differentiation are the major characteristics of stem cells [27]. In this theory, transplanted stem cells cause replacement and/or repair of injured brain cells by engraftment and replication, that may or may not have differentiation of transplanted cells into new astrocytes or microglial to develop reorganization.

Anti-inflammatory and immunomodulatory mechanisms: This is the main proposed mechanism of action showed by many of stem cells, devitalization of the inflammatory immune response to brain injury, by attenuation in the release of cytotoxins, excitotoxins, and oxygen free radicals, because of this property early application of stem cells elicit a defensive response that lessens the size and amount of white matter injury. Immunomodulatory function of MSCs is multidimensional, exhibiting not only the direct secretion of anti-inflammatory cytokines but also modifying immune cell programming and cell replication and release of pro-inflammatory cytokines, like tumor necrosis factor (TNF) from macrophages. It causes long-term systemic immune inhibition, that could be protective in the context of hypoxia-ischemia-induced neuroinflammation and perinatal brain injury [26].

Trophic mechanisms: Multitude of neurotrophic factors secreted from progenitor cells causes endogenous cell migration, proliferation, anti-apoptosis [16], differentiation, angiogenesis all of these improve cell survival. Stem cells exhibit paracrine effects to stimulate recovery in the damaged brain. Paracrine and Immunomodulatory effects on the central nervous system (CNS) microenvironment is the most restoration function in CP following the cell-based intervention, rather than direct cell replacement [18,20].

Routes and Dosage of Administration

There are multiple routes used in the transplantation of stem cells, such as intrathecal, intraspinal, intracerebral, intraventricular, intravenous, intraarterial, intramuscular, and intranasal. Type of route can be determined by many characters like the type of cells, dosage, invasive, mechanism of action of cells; there are many ideas regarding the best route. UCB was injected by intravenous, intra-arterial, intraspinal, and intrathecal. In contrast, neural stem cells (NSCs), neural progenitor cells (NPCs), and olfactory ensheathing cells (OECs) were neurosurgically delivered directly into the CNS: either the spine or the brain since NSCs, NPCs, and OECs do not cross the blood-brain barrier promptly [19]. In a study by Chen et al. using dual methods for UCB cell administrations, intra-cerebral administration did not determine a superior effect when compared to intrathecal transplantation [36]. Systemic routes may be sufficient for modulating inflammation but are likely substandard for treating local CNS lesions due to the risk of entrapment of cells in other organs and their limited ability to cross the blood-brain barrier.

Furthermore, intrathecally administered cells may not diffuse into the brain. Direct cerebral transplantation may be unfavorable in young, unstable, and medically fragile infants [20]. According to a study by Drobyshevsky, intravenous infusion of cells shows "first pass" that is cells travel through pulmonary circulation before the rest of the body, leading to high risk of cell entrapment within the lung vasculature causing pulmonary embolism therefore high mortality rates [16]. An intranasal administration of human amniotic fluid stem cells (hAFS) demonstrated neurorestorative effects in hypoxic-ischemic encephalopathy (HIE), intending it could be a beneficial treatment for HIE; however further trials should be conducted [28].

Dosage is a significant factor in the success of treatment; it should not be over or under. It should be adequate to reach the site of action [29]. A large dose of cells may cause a high risk of mortality by causing pulmonary embolization [16]. Usually, 10^7^ cells/kg body weight is used in many studies [18,29,30]. One of the studies showed even a single dosage does not show a significant difference between the treatment group and the placebo group, only post-treatment with more than or equal to 2x10^7^ cells/kg doses showed a significant difference in motor symptoms [29]. Dosage varies with the type of cell and route od administration. Adequate amount with sufficient dosing for the required period shows considerable results. Till now, most of the studies have conducted a maximum of up to 24 months [31]. Many studies assessed the outcome for a certain period of intervals, usually one, three, six, 12, and 24 months [32]. The majority of clinical trials showed a remarkable difference in motor functions according to the Gross Motor Function Measure-88 (GMFM-88) score in the treatment group than the placebo group by six months [33]. Stem cell therapy in children of less than five years of age should have better chances of improvement in motor symptoms as compared to children of more than five years because most of the gross motor development is visible at an early age [18].

The Response of Stem Cell Treatment on Various Symptoms of Cerebral Palsy

The clinical features of CP are diverse and enclose a broad range of abnormalities, predominantly motor disabilities, others like cognitive defects, language defects, hearing defects, blindness, behavioral disorders, sleep abnormalities [19]. Among all the symptoms of CP motor defects, especially spasticity is better corrected. Cognitive defects and language did not show much improvement [18]. There is a shift in the pattern of motor deficits in United States between 2006 to 2010, with an increase in the mildest and more severe form (Gross Motor Function Classification System I (38% to 48%) and V (17% to 20%), with a decrease in II (16% to 8%) and III (13% to 9%), IV was stable [20]. After six months of treatment with bone marrow mononuclear cell (BM-MNC) transplantation, QOL of CP patients is was markedly improved [34]. Table 2 shows a summary of the studies selected for the review.

**Table 2:** Studies reviewed in this article CP: Cerebral Palsy; BM: Bone Marrow; BMMSCs: Bone Marrow Mesenchymal Stem Cells; BM-MNC: Bone Marrow Mononuclear Cell; UCB: Umbilical Cord Blood; hUCB: Human Umbilical Cord Blood; NSCs: Neural Stem Cells.

Author Name and Year of publication	PURPOSE	Type of study and number of subjects included (n)	CELL TYPE	ROUTE	DOSAGE	DURATION	CONCLUSION
Himanshu et al., 2016 [31]	Autologous Bone marrow-derived stem cells for the treatment of CP	Clinical Trial. n=10	BM-derived Mesenchymal stem cells	Into subarachnoid cavity	4.5 × 10^8^ mononuclear cells; 90% viability	24 months follow up	Significant gross motor function improvement higher at month six post-treatment compared with the baseline scores and were stable up to 24 months follow-up.
Xuebin Liu et al., 2017 [35]	Comparative analysis between BM mesenchymal and BM mononuclear Stem cells in the treatment of Spastic CP	Randomized control Trail; n=105	BMMSCs BMMNCs	Intrathecal infusion	1 × 10^6^/kg body weight. Four Doses	12months	BMMSCs cells are safe and better improvement of Gross Motor function compared to BMMNCs
Than Leim Nguyen et al., 2018 [34]	Effect of BMMNCs treatment on quality of life in CP patients	Open-Label Uncontrolled Clinical Trial; n=30	BMMNCs	Intrathecal infusion	8 mL/kg for patients weighing less than 10 kg and [80 mL+(bodyweight in kg-10) × 7 mL], but not exceeding 200 mL in total, for patients weighing more than 10 kg.	12 months	There's been a noticeable improvement in the quality of life in patients with CP who were treated with BMMNC transplantation for six months period, especially improving gross motor function and muscle tone.
Jessica M. Sun et al., 2017 [29]	Effect of Autologous Cord blood on Motor function and brain connectivity in Young CP patients	Double-blind placebo control Cross Over Study; n=63	Autologous Cord Blood cells	Intravenous infusion	Single-dose 1–5 × 10^7^ total nucleated cells per kilogram of ACB,	24 months (12 months cross over)	when dosed ≥two × 10^7^ cells per kg, an IV infusion of ACB improves whole-brain connectivity and motor function in young children with CP.
Mino Kang et al., 2015 [32]	Involvement of immune response in the efficacy of cord blood cell therapy for CP	Randomized control Trial; n=36	UCB	--------	---------	Six months	In this trial, UCB alone has shown to have a good improvement in gross motor function, inducing systemic immune reactions, and anti-inflammatory changes in the brain. Motor function was directly proportional to and positively correlated by the number of UCB cells administered; Concluding that the better outcome depended on the number of cells infused.
Li Huang et al., 2018 [24]	Human umbilical stem cell infusion therapy for CP patients	Randomized Placebo Control Trail; n=54	HUCB - MSCs	Intravenous infusion	Four doses of 5 × 10^7^ cells/kg	24 months	hUCB-MSC infusion, along with basic rehabilitation, shown to be more safe and effective in improving gross motor function in children with CP compared to basic rehabilitation alone. nevertheless, it might not be realistic in improving neurologic function through replacement by hUCB-MSC infusion IV, considering the limited quantity of MSCs through the blood-brain barrier(BBB)
Liem Than Nguyen et al., 2017 [33]	Autologous bone marrow transplant for CP	Open Labeled Uncontrolled Clinical Trial; n=40	BMMNCs	Intrathecal	8 mL/kg for patients weighing <10 kg and [80 mL+(bodyweight in kg-10) × 7 mL], but not exceeding 200 mL in total, for patients weighing >10 kg. Two doses	12 months	Autologous bone marrow mononuclear cell transplantation showed to be more effective and safer for patients suffering from cerebral palsy
Guogun Chen et al.; 2013 [36]	Neural Stem Cell like Cells derived from Autologous Bone Mesenchymal Stem Cells for CP	Non-Randomized Clinical Trail; n=60	Autologous BM-NSC like cells	Subarachnoid cavity	1-2 10^7^cells	6months	NSC like cells are safe and effective for the treatment of motor deficits related to CP

Adverse Effects of Stem Cell Therapy

Possible side effects of stem cell therapy can be related to UCB infusion, allogenic cells, various causes of encephalopathy, and transmission of viral infection [30]. During the infusion of UCB, there may be early and late complications. Early complications are fever, nausea, urticaria, hemoglobinuria, anaphylactic shock, septic shock because of bacterial infection, air embolism, transfusion-related acute lung injury, and hypocalcemia associated with transfusion of a UCB. Late complications like transfusion-associated graft versus host disease [18]. Others like vomiting, seizures, headache, dermatitis, constipation [37], dimethyl sulfoxide poisoning because of an excessive cytoprotective agent, even non-diagnosed infectious agents (prions) [4], infections related encephalopathy [36]. A meta-analysis about the safety and efficacy of stem cells showed adverse effects that rarely occurred, only in four out of 135 people in the study group and only in four out of 139 people in the control group [18].

Combination Therapy

A combination of different types of stem cells might increase the efficacy of the treatment. Stem cell therapy, in combination with hypothermia, showed better results. A case study in a child with CP, and the hypoxic-ischemic insult caused by cardiac arrest is treated with the combination showed better results [38], more extensive clinical trials supporting its efficacy should be conducted. However, therapeutic hypothermia helped to improve recovery from the hypoxic insult at term; hypothermia has not yet been tested in preterm infants [33].

Limitations

Some limitations of this review were that the data was collected from the last 10 years. Only studies conducted in humans are included. Pathophysiology and mechanism of action explained in animal models are not included. We have excluded the papers that are published in other languages. There were not many randomized control trials, and only a few articles were available about stem cell therapy. For some studies, only abstract was available. Most articles are on mesenchymal cells, and limited data is available about the application of other cells for the treatment of CP.

## Conclusions

We reviewed detailed information about the possible stem cell therapies and their benefits in patients with CP. We found that immune modulation is the major mechanism of action of stem cells, and among all the types of stem cells. Autologous umbilical cord mesenchymal stem cells are safe and most effective in treatment compared to other stem cell treatments. Among all symptoms, motor symptoms are best corrected by stem cell therapy. Still, it did not show any marked improvement in treating other symptoms like sensory impairment, cognitive defects, or visual abnormalities, etc. Most of the studies are short term and used only a single stem cell type. We recommend further research and clinical trials on stem cell therapies, long-term safety and efficacy, and their combinations in treating CP.
